# Selecting cardiac magnetic resonance images suitable for annotation of pulmonary arteries using an active-learning based deep learning model

**DOI:** 10.1038/s41598-023-41228-9

**Published:** 2023-09-19

**Authors:** Werner van der Veen, Jan-Walter Benjamins, Ming Wai Yeung, Pim van der Harst

**Affiliations:** 1 Department of Cardiology, University of Groningen, University Medical Center Groningen, Hanzeplein 1, 9713 GZ Groningen, The Netherlands; 2 Faculty of Science and Engineering, University of Groningen, Groningen, The Netherlands; 3 Department of Heart and Lungs, University Medical Center Utrecht, University of Utrecht, Utrecht, The Netherlands

**Keywords:** Cardiology, Computer science, Data mining, Image processing, Machine learning

## Abstract

An increasing and aging patient population poses a growing burden on healthcare professionals. Automation of medical imaging diagnostics holds promise for enhancing patient care and reducing manpower required to accommodate an increasing patient-population. Deep learning, a subset of machine learning, has the potential to facilitate automated diagnostics, but commonly requires large-scaled labeled datasets. In medical domains, data is often abundant but labeling is a laborious and costly task. Active learning provides a method to optimize the selection of unlabeled samples that are most suitable for improvement of the model and incorporate them into the model training process. This approach proves beneficial when only a small number of labeled samples are available. Various selection methods currently exist, but most of them employ fixed querying schedules. There is limited research on how the timing of a query can impact performance in relation to the number of queried samples. This paper proposes a novel approach called dynamic querying, which aims to optimize the timing of queries to enhance model development while utilizing as few labeled images as possible. The performance of the proposed model is compared to a model trained utilizing a fully-supervised training method, and its effectiveness is assessed based on dataset size requirements and loss rates. Dynamic querying demonstrates a considerably faster learning curve in relation to the number of labeled samples used, achieving an accuracy of 70% using only 24 samples, compared to 82% for a fully-supervised model trained on the complete training dataset of 1017 images.

## Introduction

Cardiovascular disease (CVD) is the leading cause of human mortality worldwide^1^. The burden of CVD on healthcare professionals is further exacerbated by population aging and an increasing number of patients^2,3^. Considering it is estimated that up to 90% of CVD cases are preventable, there is a growing need for effective early diagnosis of CVD^4^.

In recent years, machine learning has revolutionized computer vision and image processing. Within the medical domain, various solutions have been proposed to automate image diagnosis, disease detection, characterization of pathological features in images, and clinical decision support systems for triage^5^. Medical data is abundantly available in hospitals and is often shared in large datasets for research purposes. However, the limited availability of labeled data remains a key challenge in utilizing these large-scaled datasets to train deep neural networks^6^. Labeling medical data is a labor-intensive process that requires the involvement of trained healthcare professionals, who may have limited time to address to research^6,7^. Consequently, labeling sufficient amounts of medical data has become a bottleneck in the effective development and deployment of deep learning systems for medical imaging analysis^6^.

Active learning is a semi-supervised method that aims to reduce the required numbers of labeled samples required in machine learning. In active learning, the model learns by querying unlabeled samples from a large dataset and requesting the user to label them during training. By selecting the most informative samples for consecutive training, it is theorized that the model can achieve adequate performance while relying on considerably fewer labeled data samples^8^.

Current state-of-the-art active learning techniques commonly utilize fixed scheduling of training and querying steps. However, the rate and timing at which the model’s learning plateaus may vary for each set of labeled training-data. Consequently, determining the optimal timing for a fixed scheduler becomes a critical hyperparameter, which may be chosen arbitrarily or necessitate additional experiments for optimal selection. Conducting a training round when the model’s learning has already plateaued results in inefficiency. Conversely, if the fixed time between queries is too short, the process may not have undergone a sufficient amount of iterations to effectively extract underlying patterns from the available training data before being interrupted to select a new data sample for the next training round.

In this study we propose dynamic querying, a strategy to optimize the scheduling. We demonstrate this strategy in an implementation of active learning using a dataset of short-axis cardiac magnetic resonance (CMR) scans. The primary aim of this research is to evaluate the efficiency of applying active learning to medical images and to assess if dynamic querying reduces learning time. The performance of the proposed model is compared to a model trained utilizing a fully-supervised training method. Effectiveness of the active learning approach is assessed based on dataset size requirements and loss rates. The task at hand is to classify whether presented images are scan slices that cross the widest point of the left pulmonary artery orthogonally.

## Methods

### Study population

The UK Biobank is a prospective cohort study conducted in the United Kingdom, in which over half a million participants between the ages of 40 and 69 in a community-based population^9^ were recruited. The study received ethical approval from the North West Multi-Centre Research Ethics Committee (REC reference: 16/NW/0274). All participants of the UK Biobank provided informed consent. All the study methods comply with the relevant guidelines and regulations. This research utilized the UK Biobank resource under the application number 74395. CMR scans from 1117 individuals from the UK Biobank were included in the current study. The scans were stored in DICOM media format, comprising both pixel data and metadata, such as the orientation and position of the participant within the scanner.

### Data labeling

**Figure 1:**
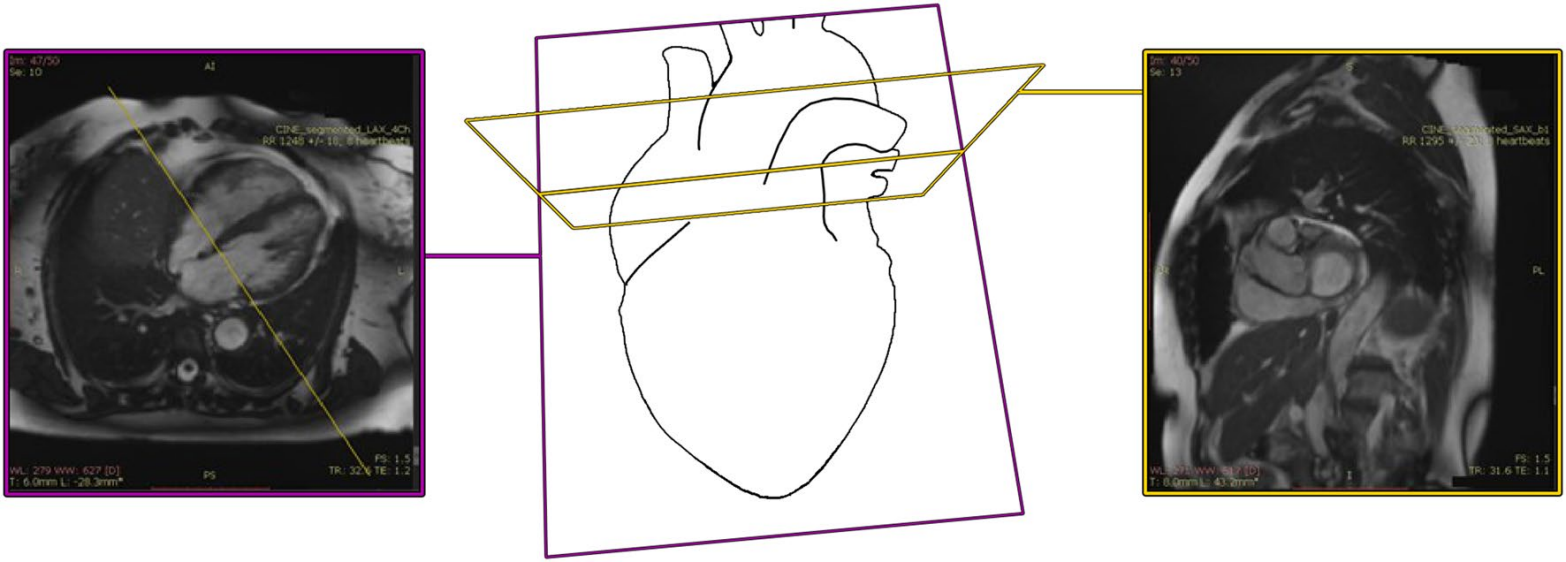
Example of a short-axis scan (right, yellow border) and its corresponding long-axis scan (left, purple border). On the left, the yellow line in the long-axis scan slice renders the projection of the short-axis scan, which is acquired perpendicularly to the long-axis scan. In the center, the orientation and location of each plane are presented schematically, also in relation to the orientation of the heart. The yellow line in the long-axis scan (left) indicates the location at which the short-axis scan (right) was obtained. Since the perpendicular, long-axis view is needed to determine the exact scan location, both scans are required to assess whether the short-axis scan is usable for annotation, making this selection process arduous and error prone. The task for the model was to classify the scans using only the information present in the short-axis scan slice.

The dataset *D* consists of two-dimensional short-axis cardiac scans (see Fig. 1, right). Each image is labeled with either the class label *included* (*I*) or *excluded* (*E*). Manual labeling was performed by a human annotator, who inspected the full three-dimensional scan volume corresponding to each scan slice. The annotator received supervision from two trained medical imaging professionals with whom all labeling decisions were discussed extensively.

**Figure 2:**
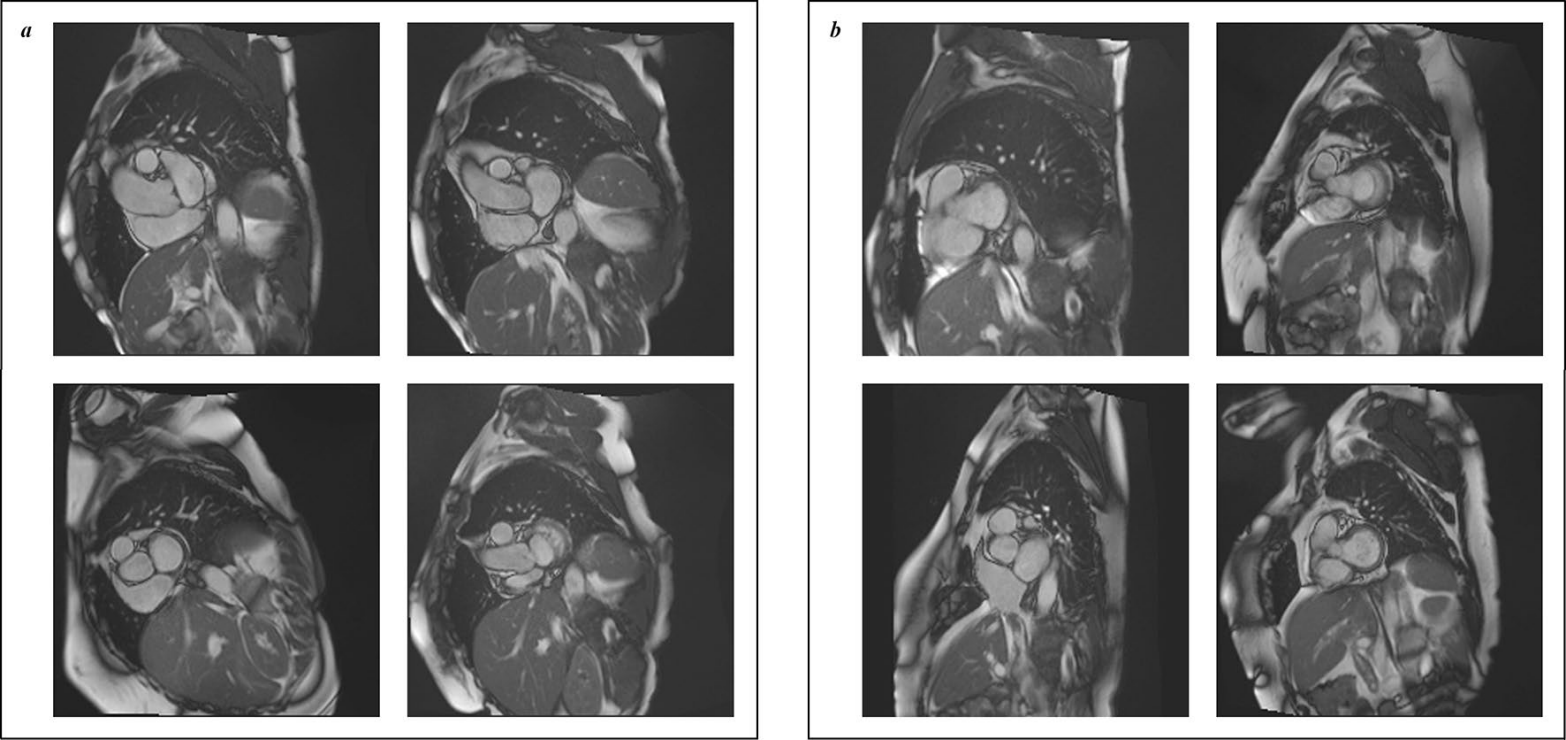
Examples of scans from the two classes. On the left (**a**), four examples of the *Included* class are presented. On the right (b), four examples of the *Excluded* class are displayed. Although clear blurs and deformations are present in some of the excluded images, some excluded images—e.g. (**b**) left-bottom—show strong resemblances with included images. Therefore, a human observer cannot classify an image for inclusion without a long-axis scan including the yellow projection line of the short-axis image (Fig. 1, left).

An image was labeled as *included* for segmentation of the pulmonary artery when it intersected the left pulmonary artery orthogonally through its widest point. This required the scan to be acquired with the appropriate orientation and positioned correctly within the participant. To assess the acquisition position, the annotator observed the short-axis scan (Fig. 1, right) in relation to the corresponding long-axis scan (Fig. 1, center and left). The position of the short-axis scan, perpendicular to the long-axis scan was indicated by a yellow line projected onto the long-axis scan (Fig. 1, left). Examples of scans labeled as *included* are presented in Fig. 2a. Images were classified as *excluded* when these criteria were not met or when the human labeler could not clearly classify due to factors such as poor image quality. Examples of scans labeled as *excluded* are displayed in Fig. 2b.

### Splitting

The two classes *I* and *E* are disjoint, meaning that no image scans exist that are elements of both classes (*E* ∩ *I* = ∅). Additionally, no other classes are present in the dataset (*D* = *E* ∪ *I*).

To evaluate the model’s performance after training, a subset of data was withheld and was not exposed to the model during training. The validation dataset *D*_*V*_ ⊂ *D* was created by randomly selecting an equal number of scans from both classes (Eq. 2) to ensure a balanced representation. Half of the smaller class was selected, with a maximum of 50 scans per class. This approach ensured that the validation set was not excessively large and allowed for an adequate number of pseudo-unlabeled samples to be queried.

1 $$\left| {D_{v} \cap E} \right| = \left| {D_{v} \cap I} \right|$$

2 $$= \max \left( {50, \frac{{\min \left( {\left| E \right|, \left| I \right|} \right)}}{2}} \right)$$

The training set *D*_*T*_ consisted of all remaining image scans after excluding the validation dataset: *D*_*T*_ = *D* \ *D*_*V*_.

A labeled dataset *D*_*L*_ was constructed by initially populating it with a preprocessed *P*(·) batch of image scans *L* randomly sampled from the training data (Eq. 3). In the active learning method employed in this paper, the size *n* of *L* was set to 1, allowing the network to select the most informative samples right after initialization. In contrast, for fully supervised learning all data points were labeled and used to train the model, *n* = |*D*_*T*_|.

3 $$D_{L} = P\left( {\left\{ {L \subseteq D_{T} \left| {card\left( L \right) = n} \right.} \right\}} \right)$$

If *n* = |*D*_*T*_|, then the system pre-labeled all data points, effectively enabling fully supervised training. On the other hand, if 1 ≤ *n* < |*D*_*T*_|, active learning was employed. In this case, some data points remained in *D*_*T*_ but were not included in *D*_*L*_, and they could be queried during the model’s learning process.

### Software

Data analyses, creation of visualizations and plots, model training and evaluation were performed in Python version 3.74. Both fully supervised and active learning neural networks were trained using Pytorch deep learning platform version 1.4.0^10^. All pixel data and metadata extractions from DICOM media files were carried out using Python package PyDicom version 1.0. Plots were generated using Python package matplotlib version 3.1.1. Further details regarding the use of Python packages and their respective versions are provided throughout the paper when relevant.

### Image preprocessing

A preprocessing pipeline *P*(·) was applied to transform the raw images into feature representations that accelerate the model training process.

Since the raw pulmonary artery scan images were large, and the predictive region of interest was presumed to be located in the approximate heart region, cropping was performed. The cropping area was empirically determined to be between ^2^*/*16 and ^10^*/*16 on the horizontal axis and between ^5^*/*16 and ^12^*/*16 on the vertical axis. Cropping not only reduced the data throughput for the model but also eliminated potentially irrelevant information for the task at hand.

Subsequently, the images were resized to 160 × 200 pixels using bilinear interpolation. Finally, the images were transformed into greyscale, resulting in one-channel encoding, and normalized to values between -1 and 1.

### Exploratory analysis

To gain insight into the difficulty of the classification task, we calculated 128 principal components from the cropped imaging data, using the method described by Tipping and Bishop^11^. Subsequently, we applied two-dimensional t-distributed stochastic neighbor embedding (t-SNE) to create a visual representation of the class distribution^12^. For both principal component analysis and for t-SNE visualization, we utilized scikit-learn Python package version 0.21.3.

### Augmentation

To mitigate the risk of overfitting, data augmentation was employed to increase the size of the training data and reduce the likelihood of overfitting. For image augmentation, we utilized Torchvision python library version 0.5.0.

In this study, we performed affine transformations that preserved potentially predictive parallel spatial patterns in the image data. The augmentation process involved randomly rotating the image clockwise or counterclockwise by at most 5°, scaling it up or down with a random factor of up to 5%, and horizontally shearing it by a random number of pixels within the range of [0,5]. These transformations effectively increased the number of training samples by generating slightly different images while retaining relevant features. Additionally, we applied two color value transformations by jittering the brightness and the contrast of the image by a factor up to 10%.

### Metadata processing

DICOM media files contain metadata—often referred to as ‘the DICOM header’—providing information about the scan, including scan dimensions, color depth, and acquisition hardware details. From each scan’s metadata, we extracted and stored information about the participant’s orientation and position relative to the scan plane during the scan. We hypothesized that this information would provide meaningful insights into the location of the short-axis scan in relation to its accompanying long-axis scan (Fig. 1). The extracted metadata values were fed to the model alongside the corresponding image pixel data. These values comprised the Cartesian coordinates (*x*, *y*, *z*), the proper Euler angles (*ϕ*, *θ*, *ψ*), two orientation vectors and a scalar value to indicate the relative position of the image slice in the full scan. Additionally, from the scout scan’s metadata—a initial scan performed by the operating technician for localization purposes—the participant’s position and orientation were used to yield combined information of the scan placement. All metadate values were normalized between 0 and 1 using the standard logistic function described in Eq. (4).

4 $$S\left( x \right) = \frac{{e^{x} }}{{e^{x} + 1}}$$

### Neural network model

#### Architecture

The model utilized in this study was a deep neural network consisting of successive, interconnected layers. The pixel data of the images was fed through a trainable ResNet18 model^13^, pre-trained on the ImageNet dataset. Transfer learning was applied between natural and medical images, assuming similarity in low-level features such as edges and shapes. The pre-trained ResNet18 model was further trained on the image data in this study.

The metadata was concatenated to the ResNet18 output, and the resulting vector was fed through two dense layers of 128 and 56 nodes, respectively. A softmax function^14^ transformed the logits into a class probability vector, which was then compared with the true label (one-hot vector) to calculate the minibatch loss. The full architecture of the neural network model is illustrated in Supplementary Fig. S1.

#### Loss

The softmax equation^14^ transformed the network’s outputs into class probability vectors, ensuring probabilities summed to 1. The *cross-entropy loss* was then calculated between these probability vectors and the “true class” vectors, represented as one-hot vectors corresponding to the image scan labels. The binary cross-entropy loss^15^, ranging from 0 to 1, indicates the dissimilarity between the two vectors.

#### Optimizer

The *stochastic gradient descent with Nesterov momentum* was employed as the optimizer function for the training the model^16,17^. This function updates the parameters in the negative direction of a gradient estimate and incorporates an additional momentum value of 0.95 to stabilize the gradient direction by accumulating current and previous gradients. This is particularly useful in handling high curvature of the loss function.

To prevent overfitting, regularization methods were employed. Weight decay—also known as L2 parameter norm penalty, ridge regression, or Tikhonov regularization^18^—was a vital regularization method in training this model, adding an L2 penalty equal to the square of the magnitude of coefficients, reducing the model’s effective capacity. In this study, a penalty value of 1 × 10^−2^ was used.

#### Learning rate scheduler

The learning rate regulates the learning speed of the network parameters and requires careful scheduling.

In this study, the learning rate was initialized at 1 × 10^−2^ and decreased by a factor of 0.9 whenever the loss on the training minibatches appeared to plateau for at least 20 epochs. This helps a more precise convergence of the model in a relatively low-error subspace within the full solution space.

#### Evaluation of classification performance

Model performance was measured using the accuracy metric, representing the percentage of correctly classified validation images. Accuracy estimates are reported with 95% confidence intervals (CI) based on model predictions in the validation holdout dataset.

### Active learning

#### Querying

In an active learning framework, images are labeled *during* the training process via queries. The querying process involves asking the oracle, such as a user in general, or an automated process in this study, to label appropriate images from the set of unlabeled images. Initial images are randomly selected, since the absence of labels in the unlabeled dataset seems to preclude more elaborate querying strategies.

#### Auto-labeling

To emulate an active learning scenario, the system utilized pseudo-unlabeled data, whose labels remained concealed until an image scan was selected during querying. This approach simulated the process of human annotation by request of the system, enabling the learning process to proceed without actual human involvement in labeling the samples during the experiment.

#### Querying strategy

We adopted a state-of-the-art querying strategy in the field of active learning, specifically the entropy-based querying strategy^19^, which falls under the umbrella of uncertainty sampling^20^.

Entropy-based sampling leverages the concept of entropy to quantify uncertainty in the model's predictions. For each unlabeled sample, the preprocessing pipeline and augmentation are applied, and the resulting sample is fed into the neural network to obtain a class probability vector, denoted as **y**. The entropy of this vector, denoted as *H* (Eq. 5), measures the distribution of the *C* different classes predicted by the model. If all classes are predicted with equal probability, the entropy value is maximal, i.e., *H* = 1. Conversely, if the class probability vector is a one-hot vector, indicating high certainty in the prediction, the entropy value is minimal, i.e., H = 0. The samples that have a high-class entropy value *H* are the most informative to the model at that point during training.

In this system, during each query, we calculated the entropy of a random selection of *E* = 500 unlabeled samples. From this selection, we queried the *L* = 1 sample(s) with the highest entropy. The parameters *E* and *L* were configurable hyperparameters, allowing for flexibility in the querying strategy. For instance, setting *E* = *L* would effectively disable the entropy test, while setting *E* = |*D*_*T*_| would lead to querying the sample(s) with the absolute highest entropy, at the cost of increased computational power when dealing with larger unlabeled datasets.

5 $$H\left( y \right) = - \mathop \sum \limits_{c = 1}^{c} y_{i} \log_{2} y_{i}$$

#### Querying scheduler

In active learning, determining the appropriate interval for querying new samples are queried is crucial to achieve the desired model performance. Querying too frequently can counteract the benefits of active learning, as the goal is to achieve high performance utilizing a minimal number of labeled samples. On the other hand, querying too sparsely can lead to overfitting and poor generalization. To address this challenge, we propose a query scheduler called *dynamic querying*, aimed at striking a balance between these extremities.

The primary objective of the query scheduler was to select new data points for labeling whenever the model’s learning from the available labeled samples became insufficient. This process was governed by two preconditions, and a new sample was queried when either of the following conditions was met:

1. If the simple moving average of the training loss of 5 most recent epochs dipped below a predefined fixed threshold of 1 × 10^−4^, or
2. If at least 20 epochs had passed since the previous query, and the training loss was plateauing. We defined the loss as “plateaued” if the mean loss of the 25% most recent epochs since the last query was higher than half of the mean loss of the preceding 75% of the epochs.

Condition 1 is implemented to prevent drastic overfitting and avoid memorizing the training data. Condition 2, on the other hand, prompted the system to select new data points when the training loss did not decrease rapidly anymore, leading the model to explore beyond a local optimum its solution space was likely in. This approach allowed the model to explore new regions in the data distribution and improve its overall performance.

To evaluate the effectiveness of dynamic querying, we conducted a comparative analysis of the model’s performance, measured by its accuracy, in relation to the number of images used in each training round. We compared these metrics between a model trained utilizing our dynamic scheduling approach, and models trained using a fixed querying schedule with epoch intervals set at 15, 25, 30, and 50.

### Hyperparameter sweep

The model training process involved several hyperparameters, including learning rate, gamma (learning rate decay factor), weight decay, batch size, and Nesterov momentum term. The same network hyperparameters were used in both the fully supervised and active learning scenario. Managing a large list of hyperparameters can be complex and time consuming, as it requires training and comparing multiple models with varying settings. To address this, we employed a hyperparameter sweep, automating the search for optimal hyperparameter combinations and reducing arbitrary choices in model design.

The hyperparameter sweeps were conducted as follows:

1. A linear search was performed for each hyperparameter, exploring a range of values, logarithmically scaled from the default settings. The best-performing value for each hyperparameter was saved, and this process was repeated three times.
2. For weight decay and initial learning rate, a grid search was performed, evaluating all combinations of the two ranges of logarithmically scaled values. The best performing combination was selected, resulting in 1 × 10^−2^ for both hyperparameters.

## Results

### Population

The dataset comprised a total of 1117 images, of which 50 images labeled as *Included* and 50 images labeled as *Excluded* were randomly selected for the validation dataset. The remaining 1017 images formed the training dataset, with 347 images labeled as *Excluded* and 670 labeled as *Included*.

### Exploratory analysis

**Figure 3:**
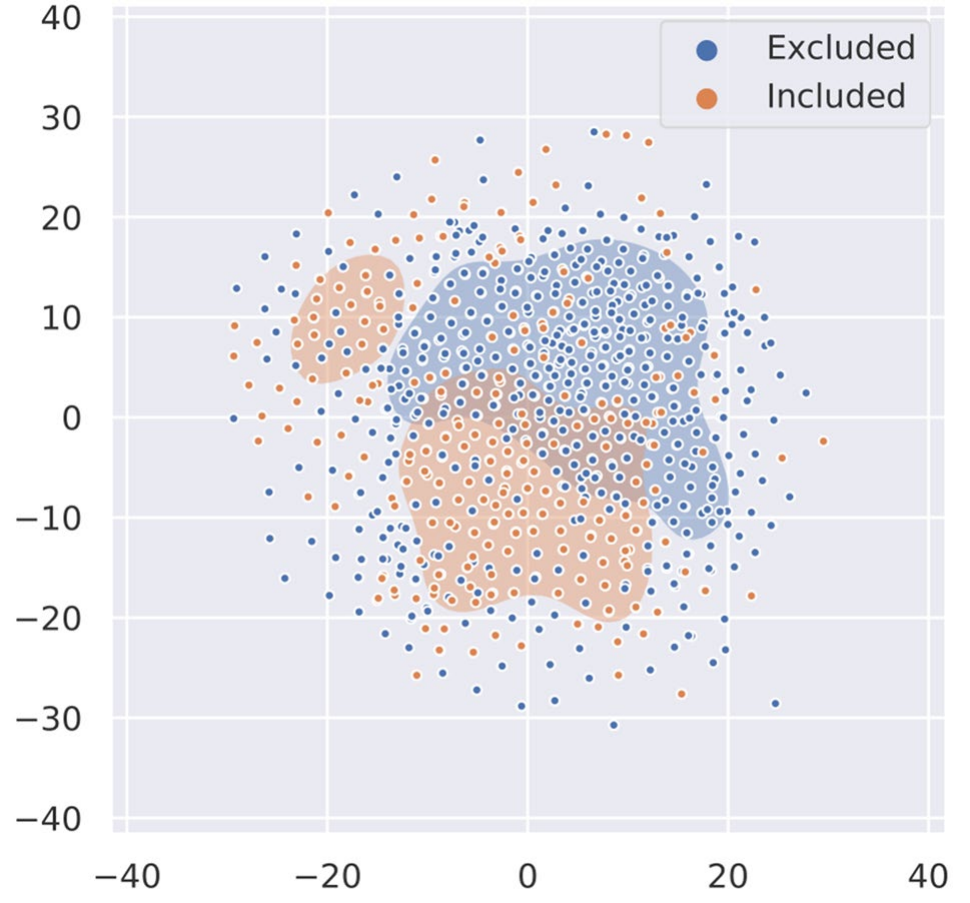
The PCA + t-SNE dimensionality reduction for the two pulmonary artery data classes, with a Kullback–Leibler divergence of 0.73.

To gain insights into the dataset's characteristics and assess its separability, we performed principal component analysis and subsequent t-SNE visualization on the preprocessed data. As Fig. 3 shows, the data comprised two weakly distinguished clusters, indicating that a moderate classification performance could be achievable on this dataset.

### Model performance

The fully-supervised method achieved an accuracy of 82% (CI 74.5–89.5%) on the complete training dataset of 1017 images, which is considered the empirical upper bound for this task given the current dataset. In contrast, the active learning achieved an accuracy of 70% (CI 63.0–77.5%) using only 24 samples after 994 epochs (CI 601–1387). While the active learning scenario naturally resulted in a lower accuracy due to the use of fewer labeled samples, it demonstrated adequate performance while requiring with significantly reduced labeling requirements (as presented in Supplementary Fig. S2).

**Figure 4:**
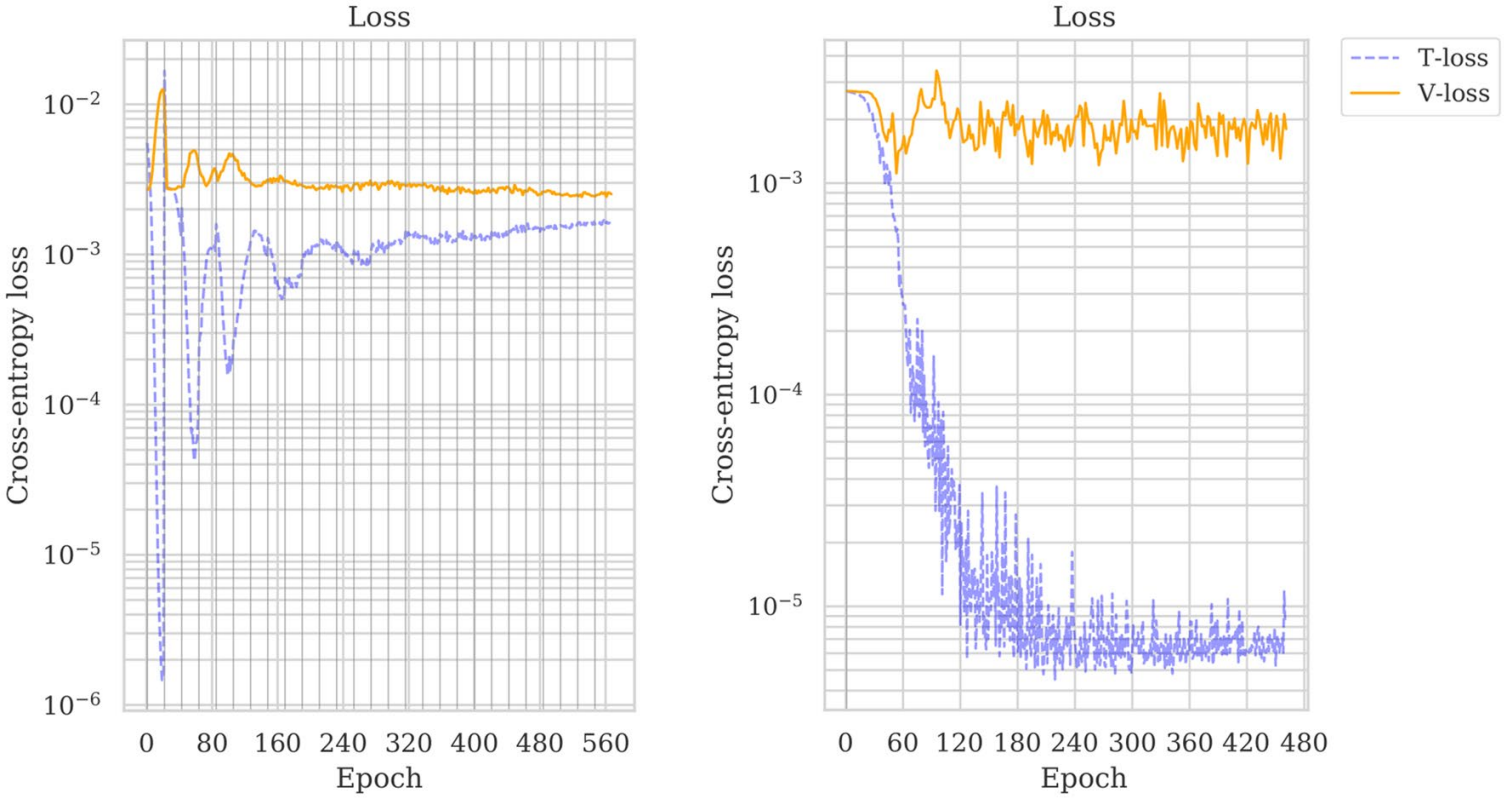
Training and validation losses (mean per minibatch). Trainings losses are indicated by blue dotted lines. Validation losses are indicated by orange solid lines. Left: losses for the active learning training run. The training loss decreases sharply until a sample is queried—indicated by vertical grey bars—after which it spikes back up. This oscillation levels off while the loss slowly increases. The validation loss follows an inverse pattern. Right: losses for the fully-supervised training run. The training loss decreases sharply and then levels off. The validation loss slightly decreases and then plateaus.

The results of the fully-supervised and active learning training runs are presented in Fig. 4 and Supplementary Figs. S2 and S3. Supplementary Fig. S2 illustrates the trade-off between the maximum validation accuracy and the number of queried samples when employing active learning.

In comparison to the dynamic querying approach, we conducted four runs with fixed querying schedules, each querying after *Q* epochs, where *Q* ∈ [15, 25, 30, 50]. To ensure a fair comparison, all runs were limited to query and select 30 samples. The run with *Q* = 15 achieved a maximum accuracy of 66% (CI 56.0–75.1%) after 908 epochs (CI 672–1144). The run with *Q* = 25 achieved a maximum accuracy of 72% (CI 65.9–77.1%) after 872 epochs (CI 530–1213). The run with *Q* = 30 achieved a maximum accuracy of 73% (CI 61.1.Y–84.6%) after 1038 epochs (CI 813–1263). The run with *Q* = 50 achieved a maximum accuracy of 68% (CI 59.4.Y–76.7%) after 1073 epochs (CI 938–1208).

To further analyze the misclassifications of the trained model, Table 1 lists four statistical measures. Notably, the numbers of false positives and false negatives were approximately equal, although such balance might not be preferred in a medical setting.

**Table 1:** Additional metrics for the fully supervised model, obtained as mean values from 50 included and 50 excluded validation scans.

Metric	Value	CI
Sensitivity	0.93	0.88–0.98
Specificity	0.92	0.87–0.97
Precision	0.90	0.84–0.96
Negative predictive value	0.95	0.91–0.99

## Discussion

The application of deep active learning on medical datasets is a promising direction of research in deep learning, but it remains relatively unexplored. This study focused on the investigation of query scheduling, an aspect of active learning that has received less attention, as compared to query selection strategies. Dynamic querying warrants further investigation, including exploring its potential combination with other existing query selection strategies, such as query-by committee^21^ or expected model change^22^. Historically, querying strategies have dominated active learning research in classical machine learning, where timing of sample selection was less crucial. This may have contributed to the relative lack of attention given to query scheduling and dynamic querying.

A fixed querying schedule is a hyperparameter and our experiments confirmed that an optimum likely exists. Hence, investigating different fixed schedules extensively is a necessity when applying fixed scheduling to achieve appropriate results. While the dynamic querying approach did not surpass the performance of the best fixed querying approach, it demonstrated comparable results and outperformed the majority of the fixed querying runs. The same holds for the number of epochs required to train a model using the dynamic scheduling. Additionally, dynamic querying exhibited greater stability in its results compared to most of the fixed querying schedules. Despite not achieving the absolute best performance, dynamic querying offers a significant advantage by enabling active learning without the need to extensively investigate and fine-tune querying schedules, while still delivering near-optimal performance. Translated to future studies, dynamic querying possesses a self-optimizing characteristic that can greatly accelerate the initiation of active learning projects.

However, it is important to note that our study focused solely on the application of dynamic querying to a specific tailored dataset for a specific purpose. Therefore, no definite conclusions can be drawn from our experiments, regarding the performance of dynamic querying on other imaging modalities, data types, or different problem domains. To fully understand the generalizability of dynamic querying, it is essential to evaluate its effectiveness across various data types and imaging modalities, thereby providing deeper insights into its potential impact on diverse research topics.

An important observation during our experiments was that the accuracy dropped after the initial query round in the active learning strategy. This phenomenon was consistent across different approaches and in line with previous findings on entropy-based querying^23^, indicating the need for improvement in the initial sample selection of the active learning algorithm. Additionally, we noticed that the initial validation accuracy varied among the different active learning strategies employed. These findings underscore the importance of refining the sample selection process to enhance the performance and stability of the active learning algorithm.

Enlarging the initialization minibatch could potentially provide a better and more predictable initial selection of the unlabeled dataset. However, this would require more human labeling, which would move the system towards ordinary supervised learning. Alternatively, other methods, such as representativeness-based approaches^24^ or adaptive methods balancing between uncertainty-based and representativeness-based selection^25^ have been previously proposed, which could be explored to sample the first image(s). These approaches might bootstrap the model with a general case before querying more uncertain samples and, importantly, offer an opportunity for follow-up studies to investigate the effectiveness of dynamic querying in combination with a more balanced selection strategy.

Larger unlabeled datasets hypothetically achieve better active learning results when using the entropy method, as queried samples are more likely to be near the decision boundary. In this study, we initially trained the active learning system on a dataset of—up to—1017 scans to achieve an initial accuracy of approximately 70% (CI 61.0–79.0%), with plans to continue training on the complete UK-biobank imaging dataset, which is an order of magnitude larger.

While the entropy-based querying strategy proved effective in many cases, the potential effects of outliers and noisy data were not explored in detail. It is possible that high-entropy data may include outliers or corrupted samples that can confound the model’s training process. It is a known concern in uncertainty sampling that selection strategies may be prone to incorrect estimations regarding the true decision boundaries, partially due to outliers^23^. Implementing a quality control preprocessing task to detect and exclude outliers could enhance the overall performance.

Regarding model selection, we opted for the ResNet18 architecture because of the seemingly difficult classification task, in which the three-dimensional context of the image slice had to be inferred from the slice itself. This decision was based on the ResNet18’s well-established performance in natural image classification. It has been suggested in prior research that employing pretrained ImageNet architectures in medical domains may be suboptimal^26^, while others assert the opposite^27^. Our study demonstrates the considerable potential of employing active learning with transfer learning on the ResNet18 architecture for the analysis of medical imaging scans, utilizing a small number of labeled images.

## Strengths and limitations

The key strength of this work lies in the introduction of a dynamic query scheduler, optimizing active learning processes by efficiently timing queries for additional labeled data. This approach minimizes the queried training data and time while maintaining model accuracy. However, there are some limitations to consider. Firstly, the dataset used in this study was labeled by a single annotator, potentially introducing observer bias that could both models’ reported performances. To mitigate this risk, the annotator was supervised by two trained medical imaging experts. Involving multiple annotators to label the dataset independently could further reduce observer bias and enhance model performance.

Secondly, the complexity of the classification problem itself may pose a limitation. Even for trained professionals, classifying the short-axis scans, without considering their relation to the corresponding long-axis images can be challenging. For this reason, the model might have learned to classify based solely on the criterion of orthogonality of the scan slice with respect to the pulmonary artery, without considering whether it was sliced through the widest point. We addressed this limitation by including extra metadata parameters. However, to further improve classification accuracy, incorporating adjacent short-axis scan slices, corresponding long-axis scans, or voxels encompassing the pulmonary artery region might be necessary. While these additions could enhance the model’s performance, they would also increase the input dimensions of the ResNet18, potentially complicating the model training and necessitating a larger-scaled training dataset.

Thirdly, the relatively small dataset of 1117 images may have limited the model’s optimal performance. However, this is a common constraint in the medical domain, and our study aimed to demonstrate an efficient training approach under such circumstances, rather than achieving the best possible performance.

## Conclusion

In conclusion, our study involved the training of two deep neural networks for the selection of short-axis CMR images suitable for pulmonary artery annotation. One model was trained using fully supervised learning on the entire dataset, while the second model was trained on a minimum of 24 images, employing active learning. We introduced a novel query timing strategy to optimize model performance with minimal labeled samples. Although the fully supervised method achieved higher accuracy compared to active learning, the latter approach demonstrated promising results by reaching peak performances with significantly fewer labeled samples, and without the necessity of prior tuning of a query scheduling hyperparameter. This suggests that active learning with dynamic query scheduling holds great potential for enhancing practicality and applicability of deep neural in cardiovascular imaging research, particularly in scenarios with limited availability labeled imaging data.

### Supplementary Information

Supplementary Information.

## Data Availability

The data that support the findings of this study are available from the corresponding author upon reasonable request. Source data from UK biobank may be requested from the UK biobank.
