# Supplementary material for: Selecting cardiac magnetic resonance images suitable for annotation of pulmonary arteries using an active-learning based deep learning model
Source: Sci Rep. 2023 Sep 19;13:15478. doi: 10.1038/s41598-023-41228-9 (PMC10509220; doi:10.1038/s41598-023-41228-9)
Supplement: Supplementary file 1 — Supplementary Information. [file 41598_2023_41228_MOESM1_ESM.pdf]

Supplemental material to:

Selecting cardiac magnetic resonance images suitable for annotation of pulmonary arteries using an active-learning based deep learning model

Werner van der Veen, MSc[1, 2], wernervdveen@gmail.com,

Jan-Walter Benjamins, BSc[1], j.w.benjamins@umcg.nl,

Ming Wai Yeung, Msc[1,3], m.w.yeung@umcg.nl,

Prof. Pim van der Harst, MD[1, 3, *], p.van.der.harst@umcg.nl,

1. University of Groningen, University Medical Center Groningen, Department of Cardiology, Groningen, The Netherlands
2. University of Groningen, Faculty of Science and Engineering, Groningen, The Netherlands
3. University Medical Center Utrecht, Department of Heart and Lungs, University of Utrecht, Utrecht, The Netherlands

Index

Supplementary figures

Figure S1. Architecture of the full neural network model

Figure S2: The samples/accuracy trade-off for the active learning training run, utilizing dynamic querying

Figure S3: Validation accuracy plots

Figure S4: Samples/accuracy trade-off plots for the active learning training runs, utilizing fixed query scheduling.

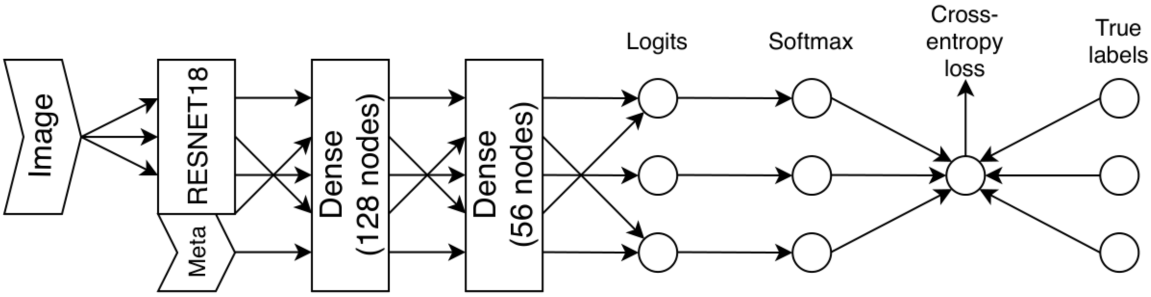

Figure S1: Architecture of the full neural network model. The image minibatch is fed through a pretrained single-channel ResNet18 and the activations of its final dense layer (of 512 units) are concatenated with the preprocessed metadata. This concatenation is processed in two more layers of respectively 128 and 56 nodes.

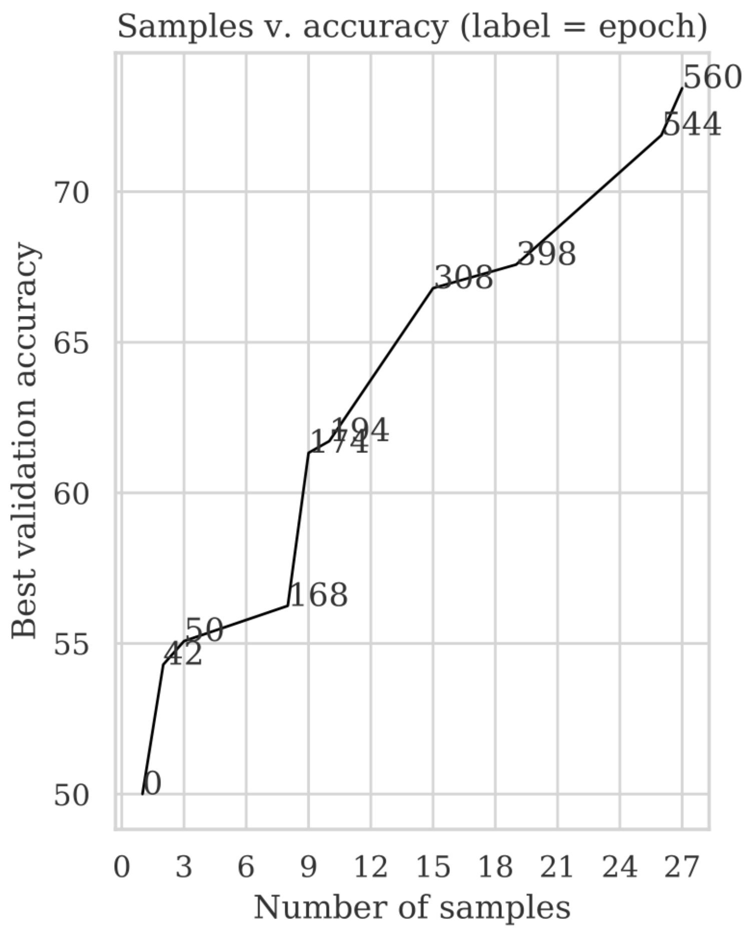

Figure S2: The samples/accuracy trade-off for the active learning training run, utilizing dynamic querying. There appears to be a linear relationship, but this is hypothesized to level off to an accuracy comparable to that reached during the fully-supervised training run.

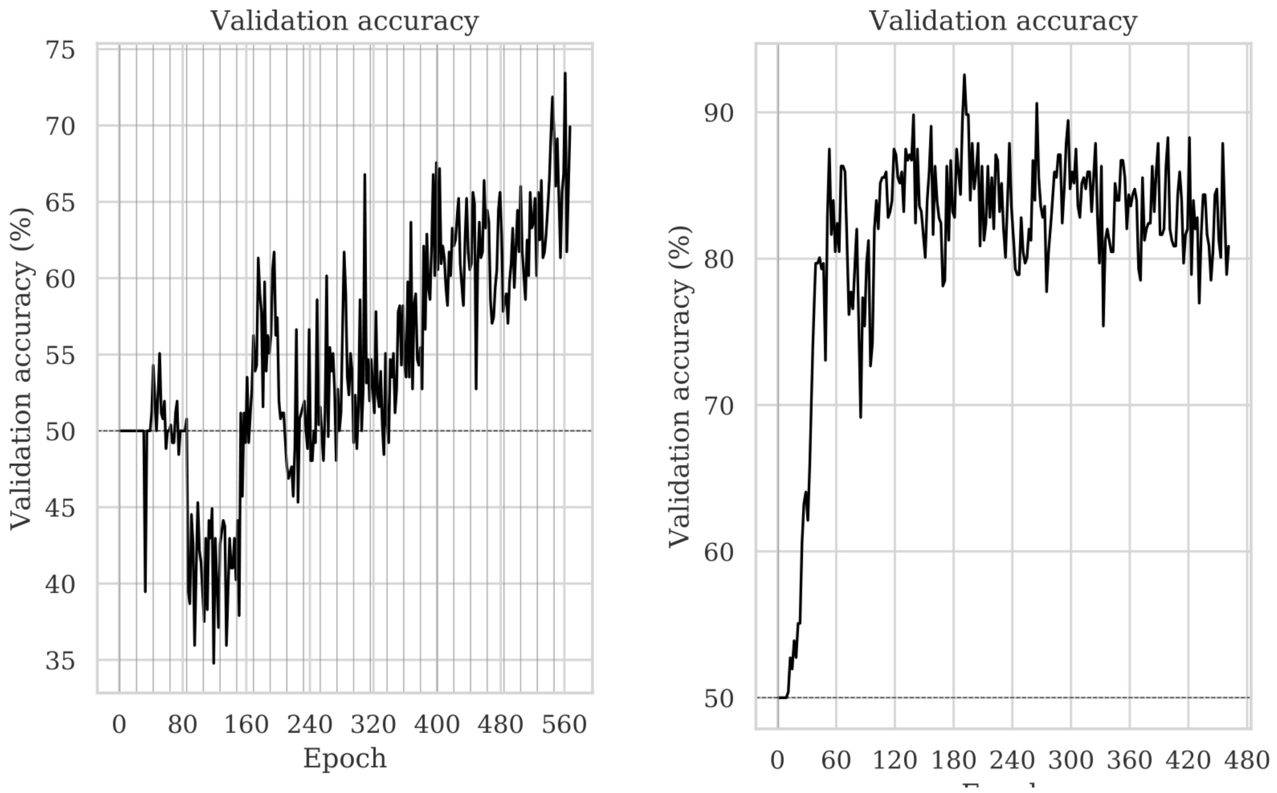

Figure S3: Validation accuracy plots. The black dotted lines indicate the baseline accuracies for binary datasets (50%). Left: validation accuracy for the active learning training run. The accuracy quickly drops and remains poor, even sub-random, until approximately epoch 200. Then it linearly increases to 60%–75% at approximately 24 image scan samples. Vertical grey bars indicate queries at respective time points. Right: validation accuracy for the fully-supervised training run. The accuracy increases sharply and plateaus between 75% and 90%, with a peak accuracy of 92%. This peak coincides with the moment that the training loss starts to plateau in Figure 7, right.

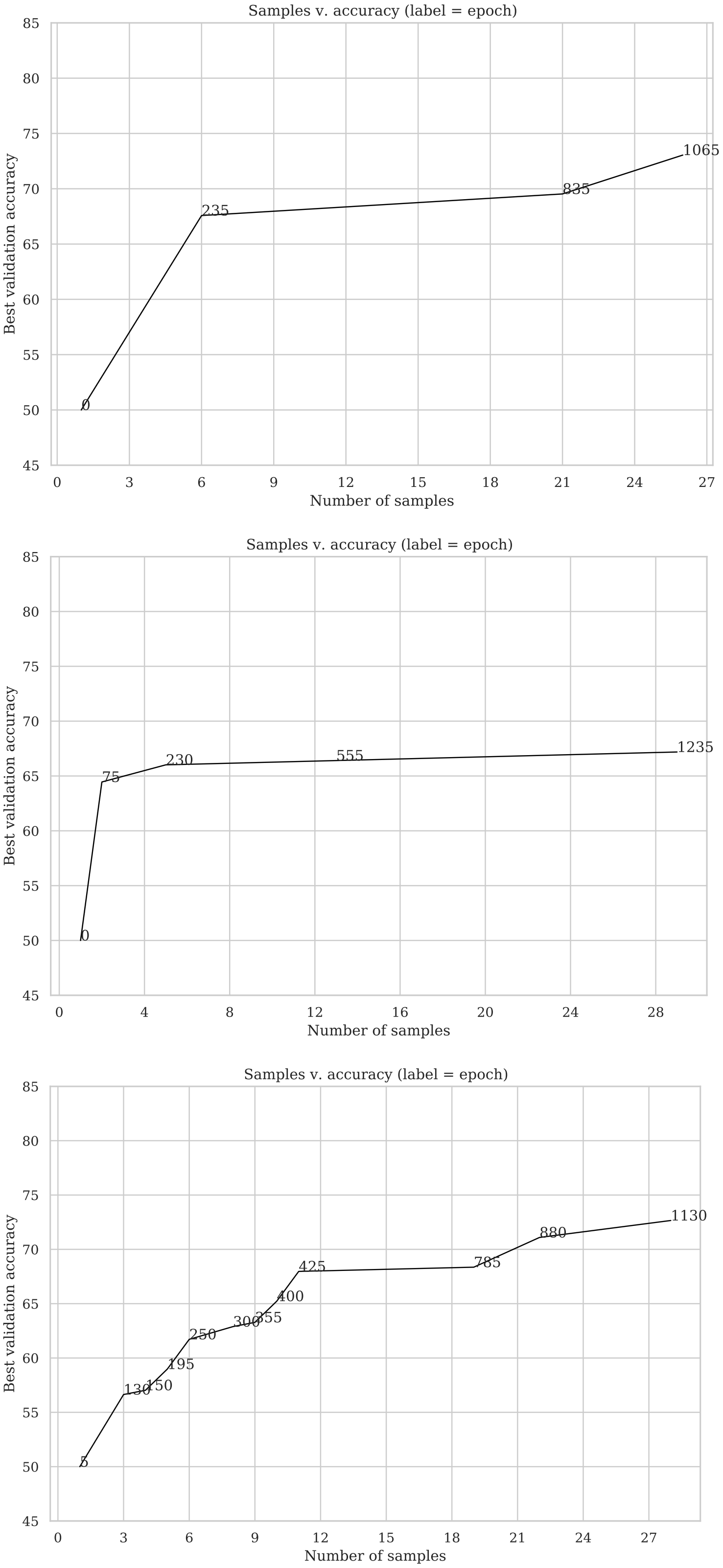

Figure S4: Examples of samples/accuracy trade-off plots for the active learning training runs, utilizing fixed query scheduling. Consecutive figures display an example of runs utilizing 25, 30, and 50 epochs between selection runs.
